# A case-based video review of arteriovenous fistulae

**DOI:** 10.3171/2025.8.FOCVID24147

**Published:** 2025-10-01

**Authors:** Jonathan J. Russin, Stavropoula I. Tjoumakaris, Mika Niemela, Erez Nossek, Jeffrey Steinberg, Sepideh Amin-Hanjani

**Affiliations:** 1 Department of Neurological Surgery, University of Southern California, Los Angeles, California;; 2 Department of Neurosurgery, Thomas Jefferson University Hospital, Philadelphia, Pennsylvania;; 3 Department of Neurosurgery, Helsinki University Hospital, Helsinki, Finland;; 4 Department of Neurosurgery, NYU Langone Health, New York, New York;; 5 Department of Neurosurgery, University of California, San Diego, California;; 6 Department of Neurosurgery, University Hospitals Cleveland Medical Center, Case Western Reserve University School of Medicine, Cleveland, Ohio

Arteriovenous fistulae are a heterogeneous collection of pathologies with presentations ranging from pulsatile tinnitus to intracranial hemorrhage. Prognosis and treatment recommendations are largely based on the presence of venous hypertension. Refined classification systems provide surgeons with a framework for clinical decision-making.^1,2^ Additional work has been done in recent years to predict the likelihood of complete obliteration using a clinically facile scoring system.^3^ Surgical treatment of arteriovenous fistulae continues to be truly multimodal, with surgical and endovascular techniques remaining relevant. This collection of videos attempts to demonstrate the breadth of presentations, treatment considerations, strategies, and techniques. Arteriovenous fistulae occur throughout the entire neuroaxis. A broad understanding of these lesions is important for all neurosurgeons. We believe that this collection of cases provides an overview that is relevant to all practicing subspecialists as well as general practitioners.

## Disclosures

Dr. Tjoumakaris reported consulting for MicroVention and Medtronic. Dr. Nossek reported consulting for Rapid Medical and Longeviti, and medical board membership for Longeviti.
